# Some New Results Involving Past Tsallis Entropy of Order Statistics

**DOI:** 10.3390/e25121581

**Published:** 2023-11-24

**Authors:** Mansour Shrahili, Mohamed Kayid

**Affiliations:** Department of Statistics and Operations Research, College of Science, King Saud University, P.O. Box 2455, Riyadh 11451, Saudi Arabia; msharahili@ksu.edu.sa

**Keywords:** order statistics, past Tsallis entropy, Shannon entropy, past lifetime, (*n*−*i*+1)-out-of-*n* structure

## Abstract

This work focuses on exploring the properties of past Tsallis entropy as it applies to order statistics. The relationship between the past Tsallis entropy of an ordered variable in the context of any continuous probability law and the past Tsallis entropy of the ordered variable resulting from a uniform continuous probability law is worked out. For order statistics, this method offers important insights into the characteristics and behavior of the dynamic Tsallis entropy, which is associated with past events. In addition, we investigate how to find a bound for the new dynamic information measure related to the lifetime unit under various conditions and whether it is monotonic with respect to the time when the device is idle. By exploring these properties and also investigating the monotonic behavior of the new dynamic information measure, we contribute to a broader understanding of order statistics and related entropy quantities.

## 1. Introduction

The mathematical study of the storage, transmission, and quantification of information is known as information theory. The field of applied mathematics lies at the intersection of statistical mechanics, computer science, electrical engineering, probability theory, and statistics. A foundational method for determining the level of uncertainty in random events is provided by information theory. Its applications are many and are outlined in Shannon’s influential work [1]. Entropy is an important parameter in information theory. The degree of uncertainty regarding the value of a random variable or the outcome of a random process is measured by entropy. For example, determining the outcome of a fair coin toss provides less information (lower entropy and lower uncertainty) than determining the outcome of a dice roll where six equally likely outcomes are obtained. Relative entropy, the error exponent, mutual information, and channel capacity are some other important metrics in information theory. Source coding, algorithmic complexity theory, algorithmic information theory, and information-theoretic security are important subfields of information theory.

Applications of the basic concepts of information theory include channel coding/error detection and correction and source coding/data compression. The development of the Internet, the compact disk, the viability of cell phones, and the Voyager space missions have all benefited greatly from its influence. Statistical inference, cryptography, neurobiology, perception, linguistics, thermophysics, molecular dynamics, quantum computing, black holes, information retrieval, intelligence, plagiarism detection, pattern recognition, anomaly detection, and even the creation of art are other areas where the theory has found application.

Probability theory and statistics form the basis of information theory, in which quantifiable data is usually expressed in the form of bits. Information measures of distributions associated with random variables are a frequent topic of discussion in information theory. Entropy is a crucial metric that serves as the basis for numerous other measurements. The information measure of a single random variable can be quantified thanks to entropy. Mutual information, which is defined as a measure of the joint information of two random variables and can be used to characterize their correlation, is another helpful idea. The first number sets a limit on the rate at which the data generated from independent samples with the given distribution can be successfully compressed. It is a property of the probability distribution of a random variable. The second number, which represents the maximum rate of reliable communication over a noisy channel in the limiting case of long block lengths, is a property of the joint distribution of two random variables when the joint distribution determines the channel statistics.

When analyzing a random variable (rv) *X* that is non-negative and has a cumulative distribution function (cdf) F(x), which is continuous, and a probability density function (pdf) f(x), the Tsallis entropy of order α is an important measure, which is elucidated in [2] as follows:

(1) $$\begin{array}{c} H_{\alpha }\left(X\right)=k_{\alpha }\left[\int_{0}^{\infty }{\left(f\left(x\right)\right)}^{\alpha }dx-1\right], \end{array}$$

where $k_{\alpha }=1/\left(1-\alpha \right)$ with α>0, α≠1. Note that $H_{\alpha }\left(X\right)=k_{\alpha }\left[E\left(f^{\alpha -1}\left(F^{-1}\left(U\right)\right)\right)-1\right]$ in which $F^{-1}\left(u\right)$ represents the right-continuous inverse of *F* and *U* is a random number (according to the uniform distribution) from the unit interval. The Tsallis entropy can yield nonpositive values in general, but appropriate choices of α can ensure non-negativity. It is worth noting that as α approaches one, H(X) converges to the Shannon differential entropy as E(−lnf(X)), thereby signifying an important relationship.

In situations involving the analysis of the random lifetime *X* of a newly introduced system, $H_{\alpha }\left(X\right)$ is commonly used to quantify the unsureness inherent in a fresh unit. Despite this, there are cases where operators know the age of the system. To be more specific, assume that they are aware that the system has been in use during an interval time with a length *t*. Then, they can calculate the amount of uncertainty in the residual lifetime after *t*, i.e., $X^{t}=\left[X-t|X>t\right]$, so that *X* stands for the original lifetime of the system. In such cases, the conventional Tsallis entropy $H_{\alpha }\left(X\right)$ does not provide the desired insight. Therefore, a novel quantity, the Tsallis entropy for the residual lifetime of the device of the lifetime unit under consideration, is introduced to address this limitation as follows:

(2) $$\begin{array}{ccc} H_{\alpha }\left(X;t\right) & = & k_{\alpha }\left[\int_{0}^{\infty }f_{t}^{\alpha }\left(x\right)dx-1\right]\\ & = & k_{\alpha }\left[\int_{t}^{\infty }{\left(\frac{f(x)}{S(t)}\right)}^{\alpha }dx-1\right], \end{array}$$

in which $f_{t}\left(x\right)=\frac{f(x+t)}{S(t)}$ represents the pdf of $X_{t}.$ The term S(t) corresponds to the reliability function (rf) of *X*. The new dynamic information quantity takes into account the system’s age and provides a more accurate measure of uncertainty in scenarios where this temporal information is available. Several recent studies have contributed to the generalization of the new measure, as discussed in Nanda and Paul [3], Rajesh and Sunoj [4], Toomaj and Agh Atabay [5], and the references therein.

Uncertainty is a pervasive feature found in various systems in nature, which is influenced by future events and even past events. This has led to the development of an interdependent concept of entropy that encapsulates the amount of uncertainty induced by incidents in the past. The past entropy is different from the residual entropy, in which the quantification of uncertainty is regarded to be influenced by events in the future. The study of entropy for past events and the relevant applications that have arisen have been accomplished by many researchers. The works carried out by Di Crescenzo and Longobardi [6] and Nair and Sunoj [7] have shed light on this topic. The research carried out by Gupta et al. [8] on the aspects and use of past entropy for order statistics was helpful in this area. In particular, they studied and performed stochastic comparisons between the entropy of the remaining lifetime of a lifespan and the entropy of the past lifetime of the lifespan, where the lifespan was quantified with respect to an ordered random variable.

Consider an rv *X* and assume it signifies the system’s lifetime. The pdf of $X_{t}=\left[t-X|X<t\right]$ is $f_{t}\left(x\right)=f\left(t-x\right)/F\left(t\right),$ in which x∈(0,t). Now, the past Tsallis entropy (PTE) as a function of *t*, the time of an observation of past failure of the system, is recognized by (see, e.g., Kayid and Alshehri [9])

(3) $$\begin{array}{ccc} {\bar{H}}_{\alpha }\left(X;t\right) & = & k_{\alpha }\left[\int_{0}^{t}f_{t}^{\alpha }\left(x\right)dx-1\right], \end{array}$$

for every t∈(0,+∞). We emphasize that ${\bar{H}}_{\alpha }\left(X;t\right)$ has a wide range of possible values, from negative infinity to positive infinity. In the context of system failures, ${\bar{H}}_{\alpha }\left(X;t\right)$ serves as a metric to quantify the uncertainty related to the inactivity time of a system, especially if it has experienced a failure at time *t*.

Extensive research has been conducted in the literature to explore Tsallis entropy’s numerous characteristics and statistical uses. For detailed insights, we recommend the work of Asadi et al. [10], Nanda and Paul [3], Zhang [11], Maasoumi [12], Abe [13], Asadi et al. [14], and the sources provided in these works. These sources provide comprehensive discussions on the topic and offer a deeper understanding of Tsallis entropy in various contexts.

In this paper, our main goal is to scrutinize the traits of PTE in terms of ordered variables. We focus on $X_{1},\ldots ,X_{n}$, as *n* identical random variables, which are independent and follow *F*. The order statistic refers to the ordering of these sample values in ascending order so that $X_{i:n}$ represents the *i*th ordered variable. These statistics have important roles in various areas of probability and statistics, as they allow for the description of probability distributions, the evaluation of the fit of data to certain models, the quality control of products or processes, the analysis of the reliability of systems or components, and numerous other applications. For a thorough understanding of the theory and applications of order statistics, we recommend the comprehensive review by David and Nagaraja [15]. The degree of predictability of an ordered random variable is usually related to its distribution; the entropy of this random variable can actually access this property. It is worth exploring the quantification of information for ordered random variables, including order statistics as a general class of statistics relevant to survival analysis and systems engineering. Aspects of information for order statistics have garnered significant attention from researchers in the literature. Several studies have explored various information properties associated with order statistics. For instance, Wong and Chen [16] demonstrated that the discrepancy among the mean entropy of ordered variables and the empirical entropy remains unchanged. They further established that, for distributions which are symmetric, the entropy of ordered variables exhibits symmetry around the median. Park [17] established some relations to acquire the entropy of ordered variables. Ebrahimi et al. [18] studied the information features of ordered random variables using Shannon entropy and the Kullback–Leibler distance. Similarly, Abbasnejad and Arghami [19] and Baratpour and Khammar [20] obtained similar results for the Renyi and Tsallis entropy of ordered random variables, respectively. Despite these efforts, the Tsallis entropy of the past lifetime of ordered variables has not been considered in literature thus far. It is commonly known that the past Tsallis entropy can be used to measure the amount of information that can be gleaned from historical observations in order to improve the forecasts of future events. This motivates us to investigate aspects of the Tsallis entropy of the past lifetime distribution of order statistics. By building upon existing research, our study aims to contribute significantly to this area by examining the behaviors of past Tsallis entropy examples for ordered variables. By highlighting previous studies and emphasizing the gap in the literature regarding the investigation of past Tsallis entropy examples in order statistics, we establish the significance and novelty of our research.

The current work’s outcomes are organized as follows: In Section 2, we derive the representation of PTE for order statistics denoted as $X_{i:n},$ which is arisen from a sample taken from an arbitrary distribution recognized by cdf F. We express this PTE on the basis of the PTE for ordered variables from a sample selected according to the law of uniform probability. We derive upper and lower bounds to approximate the PTE, since equations with exact solutions for the PTE of order statistics are frequently unavailable for many statistical models. We provide several illustrative examples to demonstrate the practicality and usefulness of these bounds. In addition, we scrutinize the monotonicity of the PTE for the extremum of a sample provided that some convenient conditions are satisfied. We find that the PTEs of the extremum of a random sample exhibit monotonic behavior as the sample’s number of individuals rises. However, we counter this observation by presenting a counterexample that demonstrates the nonmonotonic behavior of PTE for $X_{i:n}$ based on *n*. To further analyze the monotonic behavior, we examine the PTE of order statistics $X_{i:n}$ with respect to the index of order statistics i. Our results show that the PTE of $X_{i:n}$ does not change monotonically with *i*.

In what follows in the paper, the notations “$\leq_{st}$” and “$\leq_{lr}$” will be used to indicate the usual stochastic order and the likelihood ratio order, respectively. For a more detailed discussion on definitions and properties of these stochastic orders, the reader can refer to Shaked and Shanthikumar [21].

## 2. Past Tsallis Entropy of Order Statistics

Here, we acquire an expression that relates the PTE of the ordinal statistic to the PTE of an ordered random variable based on a set of values that are randomly generated according to the law of uniform probability. Let us consider the pdf and the rf of $X_{i:n}$ denoted as $f_{i:n}\left(x\right)$ and $F_{i:n}\left(x\right)$, respectively, where i=1,…,n. We have the following relationships:

(4) $$f_{i:n}\left(x\right)=\frac{1}{B(i,n-i+1)}{\left(F(x)\right)}^{i-1}{\left(S(x)\right)}^{n-i}f\left(x\right),\;x>0,$$

(5) Fi:n(x)=∑k=innkF(x)kS(x)n−k, x>0,

in which B(a,b) represents the complete beta function (see [15] for more details). Additionally, the cdf of $X_{i:n}$, i.e., the function $F_{i:n}$, is derived as

(6) $$F_{i:n}\left(x\right)=\frac{B_{F(x)}\left(i,n-i+1\right)}{B(i,n-i+1)},$$

where $B_{x}\left(a,b\right)$ represents the lower incomplete beta function. Hereafter, we shall write $Y∼B_{t}\left(a,b\right)$ to specifiy that the rv *Y* follows a beta distribution truncated on [0,t], which has density

(7) $$f_{Y}\left(y\right)=\frac{1}{B_{t}\left(a,b\right)}y^{a-1}{\left(1-y\right)}^{b-1},\;0\leq y\leq t.$$

In our context, we are concerned with the analysis of Tsallis entropy, which is measured by the cdf or pdf of the rv $X_{i:n}$. In this way, one quantifies the strength of the uncertainty induced by $\left[t-X_{i:n}|X_{i:n}\leq t\right]$ in terms of how predictable the elapsed time since the failure time of a system is. In the reliability literature, (n−i+1)-out-of-*n* structures have proven to be very useful for modeling the life lengths of typical systems. In such systems, the functionality is guaranteed only if at least (n−i+1) of the *n* units or constituents in the system are operational. A system with separate component lifetimes is headed in this way. Furthermore, a consistent distribution of the component lifetimes is assumed. The lifetime of the components in the system is denoted by $X_{1},X_{2},\cdots ,X_{n}$. The lifetime of the system is determined by the ordered rv $X_{i:n}$, where the value of *i* is the position of the order statistic. When i=1, this corresponds to a serial system, while i=n represents a parallel system. In the context of (n−i+1)-out-of-*n* structures that have experienced failures before time *t*, the PTE of $X_{i:n}$ serves as a measure of entropy associated with the past lifetimes of the system. This dynamic entropy measure provides system designers with valuable insights into the entropy of the lifetime of systems with (n−i+1)-out-of-*n* structures operating at a given time *t*.

To increase the computational efficiency, we introduce a lemma that establishes the relationship the PTE of ordered uniformly distributed rvs has with the beta function in its imperfect form. From a practical perspective, this link is essential, since it makes the computation of PTE easier. Since it only requires a few simple computations, the demonstration of this lemma—which flows immediately from the definition of PTE—is not included here.

**Lemma** **1.** *Suppose we have drawn a random sample of size n from (0,1) according to the law of uniform probability. Let we arrange the sample values in ascending order, where $U_{i:n}$ is the ith order statistic. Then,*

$${\bar{H}}_{\alpha }\left(U_{i:n};t\right)=\frac{1}{\bar{\alpha }}\left[\frac{B_{t}\left(\alpha i\bar{\alpha },1+n\alpha -i\alpha \right)}{B_{t}^{\alpha }\left(i,1+n-i\right)}-1\right],\;0<t<1,$$

 *for all* α>0,α≠1, *with* $\bar{\alpha }=1-\alpha$.

**Figure 1:**
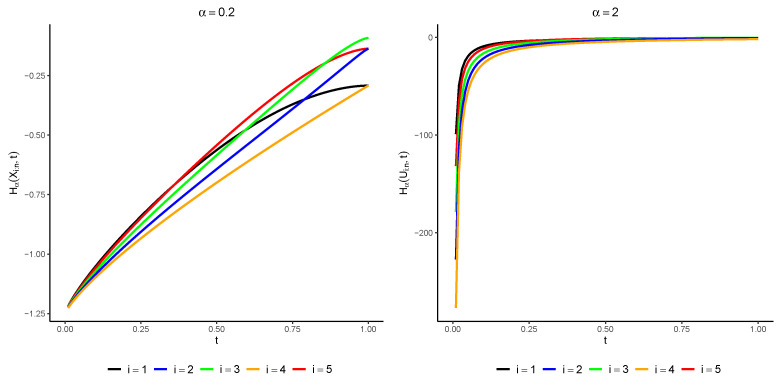
Amounts of ${\bar{H}}_{\alpha }\left(U_{i:n};t\right)$ for α=0.2 (left console) and α=2 (right console ) for various choices of 0<t<1.

This lemma provides researchers and practitioners with a useful tool to work out the PTE of the ordered variables of a sample adopted from uniform distribution. The computation can be conveniently performed via the imperfect beta function. In Figure 1, the plot of ${\bar{H}}_{\alpha }\left(Ui:n;t\right)$ is exhibited for various amounts of α, where *i* takes the values 1,2,⋯,5, and the total number of observations is n=5. The figure illustrates that there is no inherent monotonic relationship between the order statistics. The next theorem shows how the PTE of the order statistic $X_{i:n}$ is related to the PTE of the order statistic calculated for a uniform distribution.

**Theorem** **1.** *The past Tsallis entropy of $X_{i:n},$ for all α∈(0,+∞),α≠1, can be expressed as follows:*

(8) $${\bar{H}}_{\alpha }\left(X_{i:n};t\right)=\frac{1}{\bar{\alpha }}\left[\left(\bar{\alpha }{\bar{H}}_{\alpha }\left(U_{i:n};F\left(t\right)\right)+1\right)E\left[f^{\alpha -1}\left(F^{-1}\left(Y_{i}\right)\right)\right]-1\right],\;t\in \left(0,+\infty \right),$$

*so that $Y_{i}∼B_{F(x)}\left(\alpha i+\bar{\alpha },1+\alpha \left(n-i\right)\right).$*

**Proof.**  Remember that $k_{\alpha }=1/\left(1-\alpha \right)$. By making the change in variables as u=F(x), based on the formulas given in (2), (4), and (6), we obtain:

(9) $$\begin{array}{ccc} {\bar{H}}_{\alpha }\left(X_{i:n};t\right) & = & k_{\alpha }\left[\int_{0}^{t}{\left(\frac{f_{i:n}\left(x\right)}{S_{i:n}\left(t\right)}\right)}^{\alpha }dx-1\right]\\ & = & k_{\alpha }\left[\int_{0}^{t}{\left(\frac{F^{i-1}\left(x\right)S^{n-i}\left(x\right)f\left(x\right)}{B_{F(t)}\left(i,1+n-i\right)}\right)}^{\alpha }dx-1\right]\\ & = & k_{\alpha }\left[\frac{B_{F(t)}\left(\alpha i+\bar{\alpha },1+\alpha \left(n-i\right)\right)}{B_{F(t)}^{\alpha }\left(i,n-i+1\right)}\int_{0}^{t}\frac{F^{\alpha (i-1)}\left(x\right)S^{\alpha (n-i)}\left(x\right)f^{\alpha }\left(x\right)}{B_{F(t)}\left(\alpha i+\bar{\alpha },1+\alpha \left(n-i\right)\right)}dx-1\right]\\ & = & k_{\alpha }\left[\frac{B_{F(t)}\left(\alpha i+\bar{\alpha },1+\alpha \left(n-i\right)\right)}{B_{F(t)}^{\alpha }\left(i,1+n-i\right)}\int_{0}^{F(t)}\frac{u^{\alpha (i-1)}{\left(1-u\right)}^{\alpha (n-i)}f^{\alpha -1}\left(F^{-1}\left(u\right)\right)}{B_{F(t)}\left(\alpha i+\bar{\alpha },1+\alpha \left(n-i\right)\right)}du-1\right]\\ & = & k_{\alpha }\left[\left(\bar{\alpha }{\bar{H}}_{\alpha }\left(U_{i:n};F\left(t\right)\right)+1\right)E\left[f^{\alpha -1}\left(F^{-1}\left(Y_{i}\right)\right)\right]-1\right],\;t>0. \end{array}$$

The recent equality above is due to Lemma 1. This finalizes the proof. □

$$\frac{1}{\bar{\alpha }}\left[\frac{\int_{0}^{\exp(-1/t)}x^{\alpha (i-1)}{\left(1-x\right)}^{\alpha (n-i)}{\left(-\log\left(x\right)\right)}^{\alpha -1}dx}{{\left(\int_{0}^{\exp(-1/t)}x^{i-1}{\left(1-x\right)}^{n-i}dx\right)}^{\alpha }}dx-1\right]$$

Upon further calculation, it can be deduced that when the order α approaches unity in Equation (8), the Shannon entropy of the *i*th ordered variable from a set of random variables adopted from *F* can be expressed as follows:

$$\bar{H}\left(X_{i:n};t\right)=\bar{H}\left(U_{i:n};F\left(t\right)\right)-E\left[f\left(F^{-1}\left(Y_{i}\right)\right)\right],$$

in which $Y_{i}∼B_{F(t)}\left(i,n-i+1\right)$. This specific result for t=∞ has previously been derived by Ebrahimi et al. [18]. Next, we establish a fundamental result concerning the problem of monotonicity of the PTE of an rv *X*, provided that *X* fulfills the decreasing reversed hazard rate (DRHR) trait. More precisely, we say that *X* possesses the DRHR if the reversed hazard rate (rhr) function it has, i.e., the function $\tau \left(x\right)=\frac{d}{dx}\ln\left(F\left(x\right)\right)$, decreases monotonically for all x>0.

**Lemma** **2.** *If $X_{i:n}$ denotes the ith order statistic obtained from a sample following a DRHR distribution, then $X_{i:n}$ is also a DRHR.*

**Proof.**  We can express the rhr function of $X_{i:n}$ as follows:

(10) $$\tau_{i:n}\left(t\right)=\frac{f_{i:n}\left(t\right)}{F_{i:n}\left(t\right)}=h\left(\frac{F(t)}{S(t)}\right)\tau \left(t\right),\;t>0,$$

where

h(x)=xiB(i,1+n−i)∑k=innkxk,x>0.

Under the assumption that *X* is a DRHR, according to Equation (10), the distribution of $X_{i:n}$ is a DRHR if, and only if, h(x) decreases in x>0. Evidently, h(x) indeed decreases in *x*, thus completing the proof. □

We now demonstrate how the behavior of the new information measure is influenced by the DRHR feature of *X*.

**Theorem** **2.** *If X induces the DRHR feature, then the Tsallis entropy ${\bar{H}}_{\alpha }\left(X_{i:n};t\right)$ increases in t for every α∈(0,+∞).*

**Proof.**  The DRHR trait of the distribution of *X* further induces that the distribution of $X_{i:n}$ also has the DRHR trait, as stated in Lemma 2. The proof is obtained directly using Theorem 2 of the paper by Kayid et al. [9]. □

Using an example, we illustrate the application of Theorems 1 and 2.

**Example** **1.** We contemplate a distribution with the cdf $F\left(x\right)=x^{2}$ for x∈(0,1) to be the distribution of the components’ lifetimes. It is evident that $f\left(F^{-1}\left(u\right)\right)=2\sqrt{u}$ for 0<u<1. Using this information, we can derive the expression:

$$E\left[f^{\alpha -1}\left(F^{-1}\left(Y_{i}\right)\right)\right]=\frac{2^{\alpha -1}B_{t^{2}}\left(\alpha \left(i-\frac{1}{2}\right)+\frac{1}{2},1+\alpha \left(n-i\right)\right)}{B_{t^{2}}\left(\alpha i+\bar{\alpha },1+\alpha \left(n-i\right)\right)},$$

Furthermore, we can obtain:

$${\bar{H}}_{\alpha }\left(U_{i:n};F\left(t\right)\right)=\frac{1}{\bar{\alpha }}\left[\frac{B_{t^{2}}\left(\alpha i+\bar{\alpha },1+\alpha \left(n-i\right)\right)}{B_{t^{2}}^{\alpha }\left(i,1+n-i\right)}-1\right].$$

Using Equation (8), we deduce that

(11) $${\bar{H}}_{\alpha }\left(X_{i:n};t\right)=\frac{1}{\bar{\alpha }}\left[\frac{2^{\alpha -1}B_{t^{2}}\left(\alpha \left(i-\frac{1}{2}\right)+\frac{1}{2},1+\alpha \left(n-i\right)\right)}{B_{t^{2}}^{\alpha }\left(i,1+n-i\right)}-1\right],\;i=1,2,\cdots ,n.$$

**Figure 2:**
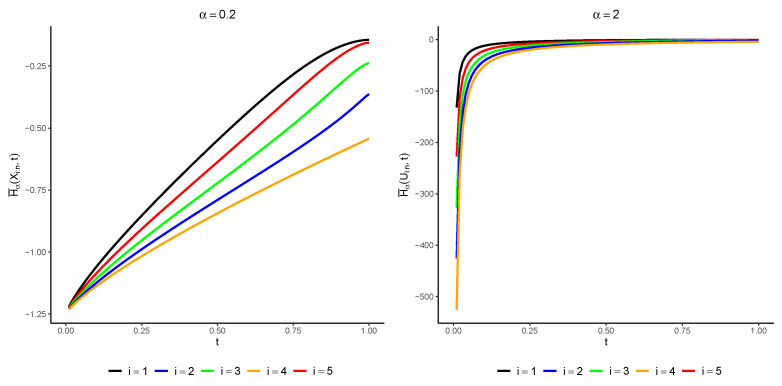
The amounts of ${\bar{H}}_{\alpha }\left(X_{i:n};t\right)$ for α=0.2 (left console) and α=2 (right console) with regard to t.

In Figure 2, we have plotted ${\bar{H}}_{\alpha }\left(X_{i:n};t\right)$ for various amounts of α with i=1,⋯,5 and n=5. It can be observed that the PTR increases with *t*, which aligns with the expectation from Theorem 2.

Unfortunately, convenient statements for the PTE of ordered rvs are not available in some situations for many distributions. Given this limitation, we are motivated to explore alternative approaches to characterizing the PTE of order statistics. We therefore propose to establish thresholds for the PTE of order statistics. To this end, we present the following theorem as a conclusive proof that provides valuable insight into the nature of these bounds and their applicability in practical scenarios.

**Theorem** **3.** *Consider a nonnegative rv X, which is continuous having pdf f and cdf F. Suppose we have M=f(m)<+∞, in which m plays the role of the mode of the underlying distribution with density F such that f(x)≤M. Then, for every α∈(0,+∞), we obtain*

$${\bar{H}}_{\alpha }\left(X_{i:n};t\right)\geq \frac{1}{\bar{\alpha }}\left[\left(\left(\bar{\alpha }\right){\bar{H}}_{\alpha }\left(U_{i:n};F\left(t\right)\right)+1\right)M^{\alpha -1}-1\right].$$

**Proof.**  Because for every α∈(1,+∞)(α∈(0,1))), one has

$$f^{\alpha -1}\left(F^{-1}\left(u\right)\right)\leq \left(\geq \right)M^{\alpha -1},$$

one can write

$$E\left[f^{\alpha -1}\left(F^{-1}\left(Y_{i}\right)\right)\right]\leq \left(\geq \right)M^{\alpha -1}.$$

The desired conclusion now clearly follows from the use of (8). This concludes the proof of the theorem. □

The recent result introduces a boundary on the PTE of $X_{i:n}$, i.e., the function which is signified by ${\bar{H}}_{\alpha }\left(X_{i:n};t\right)$. This limiting value is expressed via the PTE of the ordered variable of a set of random variables selected according to the uniform probability law and, further, the mode of the distribution under consideration, which is represented by *m*. This result yields a quantitative measure of the lower bound of the PTE with regard to the distribution mode and offers intriguing insights into the uncertainty features of $X_{i:n}$. Based on Theorem 4, we show the bound of the PTE on the ordered rvs for a few standard and reputable distributions in Table 1.

The following result establishes an upper boundary condition for the new information measure of the system with parallel structure with regard to the rhr of the distribution under consideration.

**Theorem** **4.** *Let the distribution of X fulfill the DRHR trait. For α>1, we have the inequality*

$${\bar{H}}_{\alpha }\left(X_{n:n};t\right)\leq \frac{\alpha -\tau^{\alpha -1}\left(t\right)}{\alpha (\alpha -1)},$$

*in which τ(t) is the rhr of X, which is a decreasing function by assumption.*

**Proof.**  Since the distribution of X has a decreasing rhr function, thus Theorem 2 provides that ${\bar{H}}_{\alpha }\left(X_{n:n};t\right)$ increases as t increases. Therefore, based on Theorem 3 of Kayid and Alshehri [9], we have

$${\bar{H}}_{\alpha }\left(X_{n:n};t\right)\leq k_{\alpha }\frac{\alpha -\tau_{n:n}^{\alpha -1}\left(t\right)}{\alpha }\leq k_{\alpha }\frac{\alpha -\tau^{\alpha -1}\left(t\right)}{\alpha },\;t>0,$$

in which $k_{\alpha }=1/\left(1-\alpha \right)$. Since $\tau_{n:n}\left(t\right)=n\tau \left(t\right)\geq \tau \left(t\right)$, the last inequality is easily obtained for α>1, and the proof is now complete. □

Next, we delve into the monotone behavior of the PTE of extreme order statistics with components whose lifetimes are uniformly distributed.

**Lemma** **3.** *In a system with parallel (series) structure in which components have random lifetimes following a uniform probability law, the PTE of the lifetime of the device is decreasing with respect to the components’ number.*

**Proof.**  We give the proof when the system operates in parallel. Analogous reasoning can be applied to a series system. Let us set two rvs $Z_{1}$ and $Z_{\alpha }$ with densities $f_{1}\left(z\right)$ and $f_{\alpha }\left(z\right)$, respectively, which are given by the following:

$$f_{1}\left(z\right)=\frac{z^{n-1}}{\int_{0}^{t}x^{n-1}dx}\;\mathrm{and}\;f_{\alpha }\left(z\right)=\frac{z^{\alpha (n-1)}}{\int_{0}^{t}x^{\alpha (n-1)}dx},\;z\in \left(0,t\right).$$

Next, one obtains

(12) $$\xi_{n}={\bar{H}}_{\alpha }\left(U_{n:n};t\right)=\frac{1}{\bar{\alpha }}\left[\frac{\int_{0}^{t}x^{\alpha (n-1)}dx}{{\left(\int_{0}^{t}x^{n-1}dx\right)}^{\alpha }}-1\right],\;0<t<1.$$

Let us assume that n∈[1,+∞). Then, we suppose that the derivative of $\xi_{n}$ with regard to *n* is well defined. We have the following:

$$\frac{\partial \xi_{n}}{\partial n}=\frac{1}{\bar{\alpha }}\frac{\partial \varsigma_{n}}{\partial n},$$

where

$$\varsigma_{n}=\frac{\int_{0}^{t}x^{\alpha (n-1)}dx}{{\left(\int_{0}^{t}x^{n-1}dx\right)}^{\alpha }}.$$

It is evident that for α∈(1,+∞)(α∈(0,1)):

(13) $$\begin{array}{ccc} \frac{\partial \varsigma_{n}}{\partial n} & = & \frac{\alpha A(t)}{B^{\alpha }\left(t\right)}\left(E\left[\ln\left(Z_{\alpha }\right)\right]-E\left[\ln\left(Z_{1}\right)\right]\right)\geq \left(\leq \right)0, \end{array}$$

where

$$A\left(t\right)=\int_{0}^{t}x^{\alpha (n-1)}dx,\;\mathrm{and}\;\mathrm{also}\;B\left(t\right)=\int_{0}^{t}x^{n-1}dx.$$

It is readily seen that for α∈(1,+∞)(α∈(0,1)), it holds that $Z_{\alpha }$ is greater (less) than $Z_{1}$ in usual stochastic order. Consequently, ln(z) increases as *z* grows; as an application of Theorem 1.A.3. of [21], one has $E\left[\ln\left(Z_{\alpha }\right)\right]\geq \left(\leq \right)E\left[\ln\left(Z_{1}\right)\right]$. Hence, (13) is positive (negative), and as a result, $\xi_{n}$ decreases as *n* grows. Consequently, it is deduced that the PTE of the life length of a system with parallel units decreases as the number of components increases. □

A large class of distributions consists of those that have density functions that decrease as the value increases. Some examples of these distributions are exponential, Pareto, and mixtures of distributions, among others. There are also distributions that have density functions that increase as the value increases like the power distribution. We will use the result from the previous lemma to establish the next theorem by which distributions that have density functions that are either increasing or decreasing are involved.

**Theorem** **5.** *Suppose that f is the pdf of the component’s lifetime in a parallel (series) system, and let f be an increasing (a decreasing) function. Then, the PTE of the system’s lifetime decreases as n grows.*

**Proof.**  Assuming that $Y_{n}∼B_{F(t)}\left(\alpha \left(n-1\right)+1,1\right),$ then $f_{Y_{n}}\left(y\right)$ indicates the density of $Y_{n}.$ It is evident that

$$\frac{f_{Y_{n+1}}\left(y\right)}{f_{Y_{n}}\left(y\right)}=\frac{B_{F(t)}\left(\alpha \left(n-1\right)+1,1\right)}{B_{F(t)}\left(\alpha n+1,1\right)}y^{\alpha },\;0<y<F\left(t\right),$$

increases as *y* grows. This in turn concludes that $Y_{n}$ is less than or equal to $Y_{n+1}$ in likelihood ratio order and, therefore, $Y_{n}$ is less than or equal to $Y_{n+1}$ in usual stochastic order also. In addition, α∈(1,+∞)(α∈(0,1)),$f^{-\bar{\alpha }}\left(F^{-1}\left(x\right)\right)$ increases (decreases) as *x* grows. Therefore,

(14) $$E\left[f^{\alpha -1}\left(F^{-1}\left(Y_{n}\right)\right)\right]\leq \left(\geq \right)E[f^{\alpha -1}\left(F^{-1}\left(Y_{n+1}\right)\right].$$

From Theorem 3, for α∈(1,+∞)(α∈(0,1)), one obtains

$$\begin{array}{ccc} 1+\bar{\alpha }{\bar{H}}_{\alpha }\left(X_{n:n};t\right) & = & \left[1+\bar{\alpha }{\bar{H}}_{\alpha }\left(U_{n:n};F\left(t\right)\right)\right]E\left[f^{-\bar{\alpha }}\left(F^{-1}\left(Y_{n}\right)\right)\right]\\ & \leq (\geq ) & \left[1+\bar{\alpha }{\bar{H}}_{\alpha }\left(U_{n:n};F\left(t\right)\right)\right]E\left[f^{-\bar{\alpha }}\left(F^{-1}\left(Y_{n+1}\right)\right)\right]\\ & \leq (\geq ) & \left[1+\bar{\alpha }{\bar{H}}_{\alpha }\left(U_{n+1:n+1};F\left(t\right)\right)\right]E\left[f^{-\bar{\alpha }}\left(F^{-1}\left(Y_{n+1}\right)\right)\right]\\ & = & 1+\bar{\alpha }{\bar{H}}_{\alpha }\left(X_{n+1:n+1};t\right). \end{array}$$

The initial inequality is obtained by noting that $1+\bar{\alpha }{\bar{H}}_{\alpha }\left(U_{n:n};F\left(t\right)\right)$ is nonnegative, whereas the last one is due to Lemma 3(i). Thus, we deduce that ${\bar{H}}_{\alpha }\left(X_{n:n};t\right)\geq {\bar{H}}_{\alpha }\left(X_{n+1:n+1};t\right)$ for all t∈(0,+∞). □

The following example shows that this Theorem does not work for all kinds of systems with an (n−i+1)-out-of-*n* structure.

**Figure 3:**
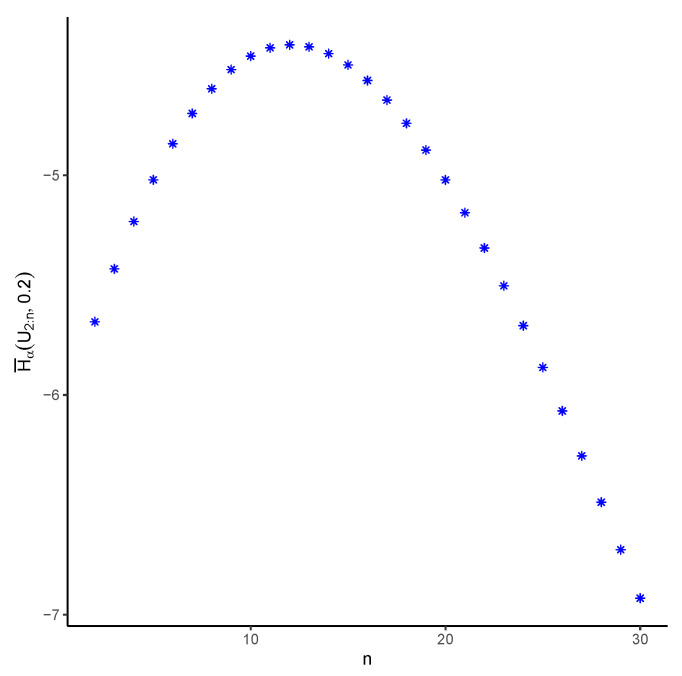
The amounts of the PTE for several choices of *n* in a system with an (n−1)-out-of-*n* structure with an underlying uniform distribution and where α=2 when t=0.2.

**Example** **2.** We presume a system is operational when more than or equal to (n−1) of the *n* components in the system are in operation. It is then not difficult to observe that the system’s random lifetime is $X_{2:n}.$ The components are assumed to have an identical distribution, which is uniform on (0,1). In Figure 3, we see how the PTE of $X_{2:n}$ changes with *n* when α=2 and t=0.2. In fact, it is observed in the graph that the PTE of the system does not always decrease as *n* increases. For example, it reveals that ${\bar{H}}_{\alpha }\left(X_{2:2};0.2\right)$ is less than that of ${\bar{H}}_{\alpha }\left(X_{2:n};0.2\right)$ for n=3,4,…,23.

## 3. Conclusions

We investigated the idea of PTE for order statistics in this paper. A novel method has been suggested by us to merge the PTE of ordered random variables belonging to a continuous distribution set with the PTE of the ordered random variables belonging to a set of random numbers selected from a uniform distribution. This relationship aids in our comprehension of PTE’s characteristics and behavior for various distributions. Additionally, because it is challenging to derive precise formulas for the PTE of order statistics, we have discovered constraints that offer helpful approximations and enable a deeper comprehension of their characteristics. The derived limits and bounds can be applied to evaluate the PTE and compare its values in different situations from different perspectives. In addition, we have investigated how the index of ordered random variables, denoted by *i*, and the number of observations, denoted by *n*, affect PTE. In order to corroborate our findings and show how our method is applicable, we included examples. These illustrations showed the usefulness of PTE for ordered random variables and the adaptability of our approach to various distributions. In short, the current work improves the perception of PTE for ordered random variables by providing the connections this quantity has with other measures, by obtaining bounds and exploring the effects of the position of the ordered variable, and by determining the impact of the size of the sample under consideration. The findings reported in this paper provide useful and profitable intuitions for professionals engaged in the analysis of information measures and statistical inferential procedures.

## Figures and Tables

**Table 1:** Lower bound on ${\bar{H}}_{\alpha }\left(X_{i:n};t\right)$ derived from Theorem 4.

pdf	Bounds
$f\left(x\right)=\frac{2}{\pi (1+x^{2})},\;x>0,$	$\geq \frac{1}{\bar{\alpha }}\left[\left(1+\bar{\alpha }{\bar{H}}_{\alpha }\left(U_{i:n};F\left(t\right)\right)\right){\left(\frac{2}{\pi }\right)}^{-\bar{\alpha }}-1\right]$
$f\left(x\right)=\frac{2}{\sigma \sqrt{2}\pi }e^{-{\left(x-\mu \right)}^{2}/2\sigma^{2}},\;x\in \left(\mu ,+\infty \right),\mu >0,$	$\geq \frac{1}{\bar{\alpha }}\left[\left(1+\bar{\alpha }{\bar{H}}_{\alpha }\left(U_{i:n};F\left(t\right)\right)\right){\left(\frac{2}{\sigma \sqrt{2}\pi }\right)}^{-\bar{\alpha }}-1\right]$
$f\left(x\right)=\frac{\lambda }{\beta }e^{-\frac{\left(x-\mu \right)}{\beta }}{\left(1-e^{-\frac{\left(x-\mu \right)}{\beta }}\right)}^{\lambda -1},\;x\in \left(\mu ,+\infty \right),\mu >0,$	$\geq \frac{1}{\bar{\alpha }}\left[\left(1+\bar{\alpha }{\bar{H}}_{\alpha }\left(U_{i:n};F\left(t\right)\right)\right){\left(\beta {\left(1-\frac{1}{\lambda }\right)}^{1-\lambda }\right)}^{\bar{\alpha }}-1\right]$
$f\left(x\right)=\frac{b^{c}}{\Gamma (c)}x^{c-1}e^{-bx},\;x>0,$	$\geq \frac{1}{1-\alpha }\left[\left(1+\bar{\alpha }{\bar{H}}_{\alpha }\left(U_{i:n};F\left(t\right)\right)\right){\left(\frac{b{\left(c-1\right)}^{c-1}e^{1-c}}{\Gamma (c)}\right)}^{-\bar{\alpha }}-1\right]$

## Data Availability

No new data were created or analyzed in this study. Data sharing is not applicable to this article.
